# Multi-view secondary input collaborative deep learning for lung nodule 3D segmentation

**DOI:** 10.1186/s40644-020-00331-0

**Published:** 2020-08-01

**Authors:** Xianling Dong, Shiqi Xu, Yanli Liu, Aihui Wang, M. Iqbal Saripan, Li Li, Xiaolei Zhang, Lijun Lu

**Affiliations:** 1 Present Address: Department of Biomedical Engineering, Chengde Medical University, Chengde City, Hebei Province China; 2 Department of Nuclear Medicine, Affiliated Hospital, Chengde Medical University, Chengde City, China; 3 Faculty of Engineering, Universiti Putra Malaysia, Serdang, Malaysia; 4 School of Biomedical Engineering and Guangdong Provincal Key Laboratory of Medical Image Processing, Southern Medical University, Guangzhou, China

**Keywords:** Deep learning, Multi-view, Medical image, Three-dimensional segmentation, Secondary input, Residual block

## Abstract

**Background:**

Convolutional neural networks (CNNs) have been extensively applied to two-dimensional (2D) medical image segmentation, yielding excellent performance. However, their application to three-dimensional (3D) nodule segmentation remains a challenge.

**Methods:**

In this study, we propose a multi-view secondary input residual (MV-SIR) convolutional neural network model for 3D lung nodule segmentation using the Lung Image Database Consortium and Image Database Resource Initiative (LIDC-IDRI) dataset of chest computed tomography (CT) images. Lung nodule cubes are prepared from the sample CT images. Further, from the axial, coronal, and sagittal perspectives, multi-view patches are generated with randomly selected voxels in the lung nodule cubes as centers. Our model consists of six submodels, which enable learning of 3D lung nodules sliced into three views of features; each submodel extracts voxel heterogeneity and shape heterogeneity features. We convert the segmentation of 3D lung nodules into voxel classification by inputting the multi-view patches into the model and determine whether the voxel points belong to the nodule. The structure of the secondary input residual submodel comprises a residual block followed by a secondary input module. We integrate the six submodels to classify whether voxel points belong to nodules, and then reconstruct the segmentation image.

**Results:**

The results of tests conducted using our model and comparison with other existing CNN models indicate that the MV-SIR model achieves excellent results in the 3D segmentation of pulmonary nodules, with a Dice coefficient of 0.926 and an average surface distance of 0.072.

**Conclusion:**

our MV-SIR model can accurately perform 3D segmentation of lung nodules with the same segmentation accuracy as the U-net model.

## Introduction

The American Cancer Society estimated that, in 2018, lung cancer remains the leading cancer type in 1.73 million new cancer patients, and hundreds of thousands of patients die of lung cancer every year [1]. CT is the most commonly used modality in the management of lung nodules and automatic 3D segmentation of nodules on CT will help in their detection and follow up. Accurate segmentation and positioning of computer-assisted 3D lung nodules can help the discovery and treatment of lung nodules and prerequisites for liver and tumor resection [2]. Recent research has shown that convolutional neural networks (CNNs) can automatically learn the characteristics of medical images, and thus can be applied in segmenting medical images with high accuracy [3–5]. Manual marking of each patient’s lesion location by physicians and radiologists is generally accepted as the gold standard for medical image segmentation. However, because the number of 3D image slices generally reaches up to several hundreds, the calibration process is time consuming and experts face immense workload due to shortage of experienced physicians and radiologists. Moreover, With the continuous development of medical technology, people are more and more concerned about their health, resulting in a significant increase in the number of CT every year. The burdens of doctors and radiologists are getting heavier, and patients have to wait longer for results, which is not conducive to the healthy development of medical and health services. The development of computer-aided intelligent segmentation classification of 3D medical images improves the processing speed of medical images, enhances the accuracy of diagnosis of diseases by doctors, and reduces the burden of physicians and radiologists [6]. The combination of artificial intelligence deep learning and medical image 3D segmentation can more accurately perform 3D segmentation of lung nodules, which is helpful for doctors to find and follow up lung nodules. CNNs have currently made great progress in 2D segmentation of medical images, but their application in 3D segmentation is still a challenging task. The reasons for this difficulty are as follows. First, the learning process of CNNs requires a large amount of 3D medical image data and their ground truths to produce good prediction results; however, there is still a lack of such large amount of data [7, 8]. Second, the class balance between negative and positive samples in a 3D dataset is a challenge. In general, there are far more negative non-nodular samples than positive nodular ones. For example, in lung CT images, some lung nodules are only 3–5 mm in diameter with extremely low volume [9]. Therefore, if a deep-learning CNN is provided with sufficient training data and a better class balance, the loss function of the CNN can be easily minimized and a good model can be effectively trained [10]. 3D CNNs consume considerable amount of computing resources, such as graphic cards and memory, during training. In the model prediction process, the trained network requires high hardware requirements and has certain restrictions on the promotion of its application. Therefore, the algorithm needs to be optimized to render it simple and dexterous such that it is more conducive to 3D medical image segmentation tasks [11].

In this study, we propose a multi-view secondary input residual (MV-SIR) model for 3D segmentation of pulmonary nodules in chest CT images. We extract lung nodules into voxel cubes, adding 10 pixels in each of the six directions of the nodule to include additional non-nodule tissues inside. After that, extract a certain amount of the voxel points in the lung nodule part, and extract equal numbers of voxel point in the expansion part to balance the positive and negative samples. In the lung nodule cube, scale patches in the axial, coronal, and sagittal views are extracted centered on randomly selected voxel points. Selecting a part of the voxel points randomly in the lung nodule cube can easily and efficiently capture most of the image features of the lung nodules, avoiding that most voxel points are too close together, which causes the extracted patches to be too similar and data redundancy. Each view extracts voxel heterogeneity (VH) features and shape heterogeneity (SH) features. The density and shape of tumor tissue are quite different from those of normal tissue, and there is a high correspondence between the judgment of nodules and their heterogeneity. In CT images, VH reflects gray-scale heterogeneity and tissue density information, and SH reflects tissue shape information. And then we construct an SIR submodel for feature learning for two patches of each view; thus, six submodels are constructed. Then, we integrate the six SIR submodels into the MV-SIR model and learn whether the patches extracted at each point in the cube belong to the pulmonary nodules. Overall, the proposed MV-SIR model has the following contributions:

1. To the best of our knowledge, it is the first time a combination of the secondary input and residual block is added to a CNN model of segmentation of CT images of 3D pulmonary nodules. This combination can provide reference for the application of the CNN model in medical image classification and segmentation tasks.
2. Using multi-view (axial, coronal, and sagittal) and multi-image (VH and SH) features as input to the MV-SIR model, full feature extraction can be performed on 3D CT medical images, which improves the accuracy of 3D lung nodule segmentation.
3. Integration of six SIR submodels from three views to one model improves the performance of the model. The model thus constructed has faster prediction speed and consumes lower equipment computing power than the 3D segmentation model of convolutional kernels.

## Related work

In recent years, an increasing number of studies have developed artificial intelligence deep learning CNN tools in the field of medical image segmentation classification [12, 13]. In 2D CNN models, a 3D medical image is sliced into 2D images for feature learning, and then 3D medical image segmentation is performed on the basis of the prediction result of the 2D CNN model [14–16]. Wang et al. captured detailed texture and nodule shape information using a scale patch strategy as the input to the MV-CNN and obtained segmentation results with an average surface distance (ASD) of 0.24 [17]. Xie et al. decomposed 3D nodules into nine fixed views to learn the characteristics of 3D pulmonary nodules, and the segmentation result of the model had an accuracy of 91.60% [18]. Another method treats a 3D image as a series of 2D slices and learns the 2D slices through a CNN model to segment the image [19]. Christ et al. serially connected two fuzzy neural network (FNN) models as the region-of-interest (ROI) input of the second FNN, and segmented the liver and its lesions. The results showed that the liver and lesion segmentation of the model had a Dice score of greater than 94% [20]. Tomita et al. extracted the radiological features of each CT image using a deep CCN and integrated them into an evaluation system. The segmentation results had an accuracy of 89.2% [21]. Furthermore, Ronneberger et al. used a U-net model to achieve high-speed end-to-end training with limited images, which provided excellent segmentation results [22]. Jonathan et al. established a “completely convolved” network that accepts inputs of any size and produces outputs of corresponding size through effective reasoning and learning [23]. In another method, 3D volume segmentation of medical images is directly performed by inputting 3D medical images into a 3D depth learning model to learn [24–27]. For volumetric image segmentation, Çiçek et al. introduced a 3D U-net model, which learns from sparsely annotated volumetric images [28]. Milletari et al. proposed a 3D image segmentation method, V-net, based on a volumetric fully convolutional neural network, to achieve end-to-end training and learning, which enable prediction of the entire volume [29].

## Method

The implementation of the proposed MV-SIR model involves the following procedures: (1) Extract lung nodule cubes from the Lung Image Database Consortium (LIDC) and Image Database Resource Initiative (IDRI) (LIDC-IDRI) CT dataset and extract patches from the three views by taking a voxel point in the cube as the center. (2) Extract VH and SH features from the slices of lung nodules. (3) Build the SIR submodel and train it with the patches extracted from the three views. (4) Combine the six branches of the lung nodule into the MV-SIR model, obtain the training results, and perform 3D reconstruction on the image segmentation results.

### Dataset and multi-view patch extraction

**Fig. 1:**
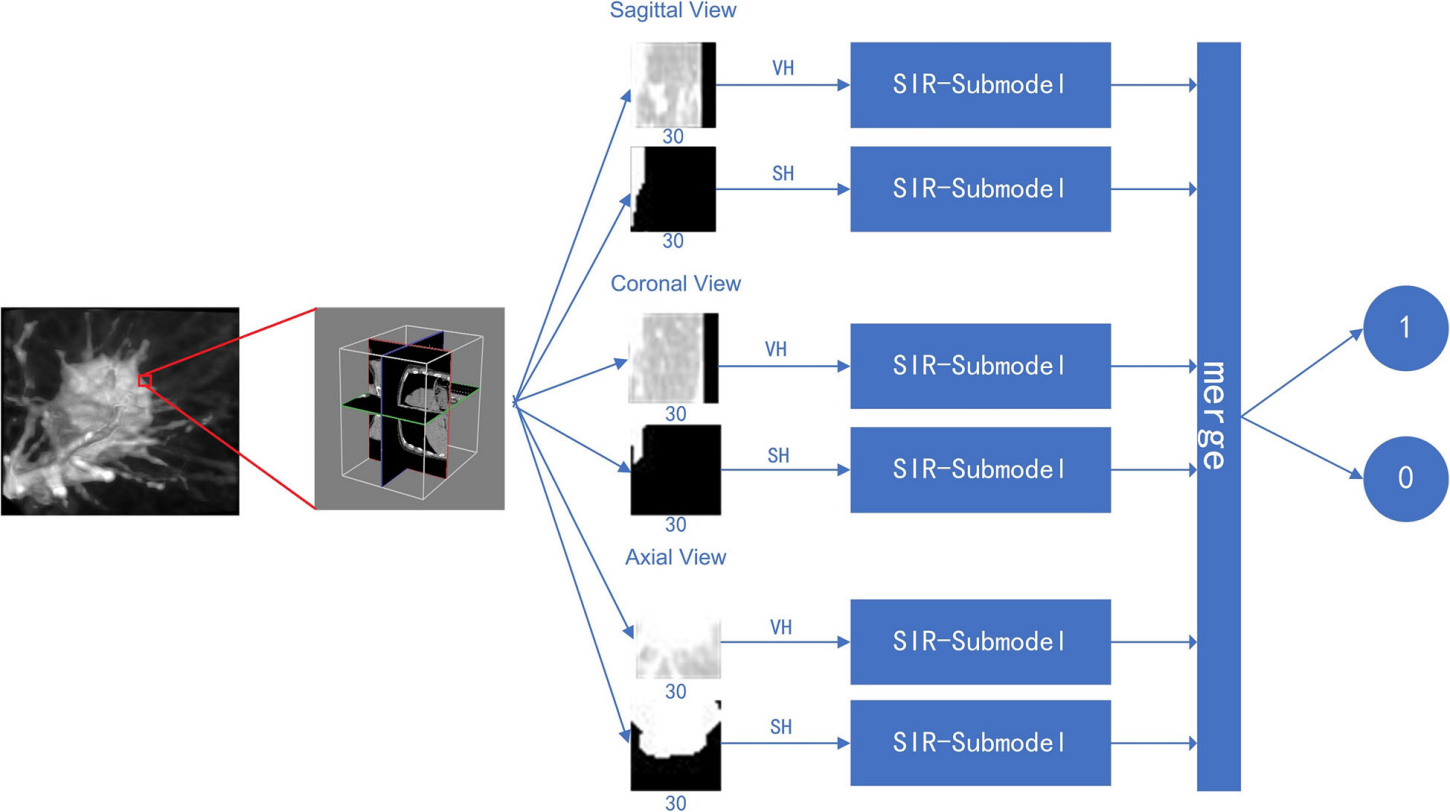
Structure of the MV-SIR model and the model training process

The LIDC-IDRI dataset was collected by the National Cancer Institute to study early cancer detection in high-risk populations. The LIDC-IDRI dataset is composed of chest medical image files (such as CT images, X-ray films) and corresponding diagnostic result lesions. A total of 1018 research samples were included in the dataset. For each of the images in the sample, a two-stage diagnostic labeling was performed by four experienced chest radiologists. In the first stage, each radiologist independently diagnosed and labeled the patient’s nodule location, categorized as follows: 1) > =3 mm nodules, 2) < 3 mm nodules, and 3) > =3 mm non-nodules. In the second stage, each radiologist independently reviewed the comments of the remaining three radiologists and determined that there were no errors, and then gave their own final marking results. The results of the four radiologists are recorded in LIDC-IDRI. In this paper, we use the average results of the four radiologists as the marked area of ​​the lung nodules. Such a two-stage annotation can mark all results as completely as possible while avoiding forced consensus. We selected a total of 874 clearly marked lung nodules, 600 lung nodules were used for model training and validation, of which the validation set accounted for 10%, and 274 lung nodules were used for model testing. All study samples were processed in the same way, so use LIDC-IDRI-0001 as an example., which is a matrix of 133 × 512 × 512, a total of 133 slices, each of size 512 × 512. According to the spatial resolution of the chest CT scan, we resampled the pixel values ​​into voxels based on the standard size of 1.0 mm × 1.0 mm × 1.0 mm, and finally obtained the voxel cube of 133 × 512 × 512 mm^3^ to complete the 3D reconstruction of CT images [30]. We extracted the nodules from the entire CT image based on the center position of the nodule and the ROI provided by the radiologists. We prepared a lung nodule cube consisting only of voxel grayscale values and added 10 voxels in the six directions of the cube, namely, top side 、bottom side、front side 、back side、left side、right side to balance the class between negative and positive samples. Although the obtained lung nodules have different cube sizes, the 2D patches we extracted are the same and uniform, which does not affect the training of our model. We extracted multi-view patches centered on a random voxel in the lung nodule cube from axial, coronal, and sagittal views. Research indicates that the best slice size is 30 × 30 [21]. Figure 1 presents the voxel points randomly selected as the center and 30 × 30 slices extracted around them in the axial, coronal, and sagittal views.

### VH and SH extraction

As 3D image segmentation needs to be converted into 2D image classification, we must classify the patches extracted from the lung nodule cubes into nodules and non-nodules before the model is used for classification training. Based on the ROI marked by the radiologist on the CT image, we obtain a polygon with the lung nodule boundaries. To judge whether a patch belongs to the lung nodule, we only need to distinguish whether the randomly located patch point is inside the polygon. This is determined by the ray method wherein a ray is drawn from the center point and the number of intersections the ray makes with the boundaries of the polygon is calculated. If the number of intersection points is odd, the point is inside the polygon, otherwise the point is outside the polygon. The patches that belong to the pulmonary nodules are denoted as 1, while those that do not belong to the pulmonary nodules are denoted as 0. On each slice, 4000 patches are extracted, with each lung nodule obtaining a patch of 4000 × m; the total number of patches is obtained as

1 $$ \boldsymbol{Patches}=\sum \limits_{\boldsymbol{i}=\mathbf{1}}^{\mathbf{n}}\mathbf{4000}\ast {\boldsymbol{m}}^{\left(\boldsymbol{i}\right)}, $$

where ***m*** represents the number of layers of lung nodules and ***n*** is the total number of lung nodules extracted. In this way, we can select one quarter to one half of the pixels in the lung nodule cube, and the extracted 2D patch can contain most of the information of the lung nodule.

As Fig. 1 shows, the extraction of VH and SH features is based on the voxel values of the CT images and the ROI calibrated by the radiologists. VH is represented by the difference in the grayscale values of the voxels and can be directly obtained from the lung nodule cube. SH is represented by different shapes. We convert the voxel grayscale value image into a binary image based on the ROI, which better reflects its shape feature.

### SIR submodel

**Fig. 2:**
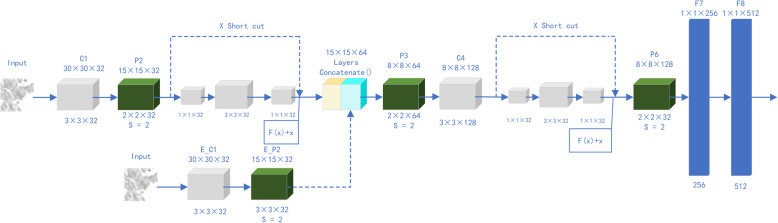
Structure of the MV-SIR submodel, and the submodel training process

SIR submodels are composed of two residual blocks and one secondary input block and are connected with several fully connected layers, pooled layers, and convolution layers. As shown in Fig. 2, the 30 × 30 image is first input into convolutional layer C1 and pooled layer P2. Convolutional layer C1 contains 32 × 3 × 3 convolution kernels, and 30 × 30 × 32 feature maps are obtained. Then, the feature map is input into P2 with 2 × 2 kernels and a step size of 2 × 2; 15 × 15 × 32 characteristic maps are obtained.

Next, in an identical residual block, the upper path is the “shortcut path” and the lower path is the “main path”. The “upper path” belongs to the shortcut of the residual block, The “main path” is the main structure of the model. The “main path” includes the first convolution layer with a filter size of 1 × 1, a step size of 1 × 1, and padding = “valid” is no fill convolution; the second convolution layer with a filter size of 3 × 3, a step size of 1 × 1, and padding = “same” is the same convolution; and the third convolution layer with a filter size of 1 × 1, a step size of 1 × 1, and padding = “valid” is no fill convolution. The shortcut path is to input information by shortcut to the module with the “Layeradd” function, and then the ReLU activation function is applied. The constant residual block input and output are the same, so a 15 × 15 × 32 feature map is obtained. The residual block protects information integrity by directly passing the input information to the output. The entire network only needs to learn the input and output differences, thus simplifying the learning objectives and complexity. This process is conducive to improve the efficiency of CNN learning; the specific improvement principle will be analyzed in detail in the discussion.

The secondary input is made via another path. After the original image is passed through a few convolution operations, it is stitched to the output of the first residual block using the concatenate function. Note that, as shown in Fig. 2, the “Layeradd” function is different from the “Layerconcatenate” function. The former directly adds the value of one matrix to another matrix, and the resulting matrix dimensions are unchanged, although the values ​​of the matrix change. The latter function changes the dimensions of a matrix by splicing one matrix onto another, keeping the values ​​of the matrix unchanged. The 30 × 30 image is input into convolutional layer EC1 and pooled layer EP2, and 15 × 15 × 32 secondary characteristic maps are obtained. After splicing of the matrix using the “Layerconcatenate” function, we obtain a 15 × 15 × 64 feature map matrix as the input to the subsequent layer.

Next, the image is input to pooling layer P3 and convolution layer C4. Because our image size is small, in order to better preserve the integrity of the image information, the image is input again into a constant residual block and a pooling layer, and an 8 × 8 × 128 feature graph matrix is obtained. Finally, the image is sequentially input to two 1 × 1 × 256 fully connected layers, F7 and F8. This completes the construction of our secondary input residual (SIR) submodel.

### MV-SIR model

As shown in Fig. 1, the MV-SIR model is composed of six SIR submodels. The submodel inputs are VH and SH patches from axial, coronal, and sagittal views. In each lung nodule cube, 6 × 4000 × m patches are input to the MV-SIR model. We extracted 600 pulmonary nodules for training 274 lung nodules for testing. The total number of patches is given:

2 $$ \boldsymbol{All}-\boldsymbol{Patches}=\sum \limits_{\boldsymbol{i}=\mathbf{1}}^{\mathbf{674}}\mathbf{4000}\ast \mathbf{6}\ast {\boldsymbol{m}}^{\boldsymbol{i}}, $$

A fully connected layer fuses all submodels and is connected to the classification layer of a neuron. The activation function of the output layer of a neuron is a two-class problem, so we use the classical Sigmoid function, given as follows:

3 $$ \boldsymbol{\mathsf{\boldsymbol{\delta}}}\left(\boldsymbol{z}\right)=\frac{\mathbf{1}}{\mathbf{1}+{\boldsymbol{e}}^{-\boldsymbol{z}}}\in \left[\mathbf{0},\mathbf{1}\right],\boldsymbol{z}\in \left(-\infty, +\infty \right), $$

where ***z*** is the output of the model. For the loss function, binary_cross_entropy, which is a binary entropy class, is selected. There are only two types, 0 or 1, which can overcome the problem that the variance cost function update weight is too slow [31]. The loss function ***L*** is given by the following formula:

4 $$ \boldsymbol{L}=-\frac{\mathbf{1}}{\boldsymbol{n}}\sum \limits_{\boldsymbol{i}=\mathbf{1}}^{\boldsymbol{n}}\left[{\boldsymbol{y}}^{\left(\boldsymbol{i}\right)}\boldsymbol{\log}{\hat{\boldsymbol{y}}}^{\left(\boldsymbol{i}\right)}+\left(\mathbf{1}-{\boldsymbol{y}}^{\left(\boldsymbol{i}\right)}\right)\boldsymbol{\log}\left(\mathbf{1}-{\hat{\boldsymbol{y}}}^{\left(\boldsymbol{i}\right)}\right)\right], $$

Here, **y**^(**i**)^ is the true result of the calibration and ${\hat{\boldsymbol{y}}}^{\left(\boldsymbol{i}\right)}$ is the model prediction result. We use the adaptive learning rate optimization method Adam to calculate the adaptive learning rate of each parameter. Practical application of Adam has demonstrated that it is better than other adaptive learning methods in that it is simple to implement, is efficient in calculation, consumes less memory, is extremely interpretative, and usually requires no adjustments or only minor fine-tuning as well as the parameter update in the algorithm is not affected by gradient transformation [32]. The learning rate and weight decay are 0.0001 and 0.01, respectively, and the batch size is 2000.

**Fig. 3:**
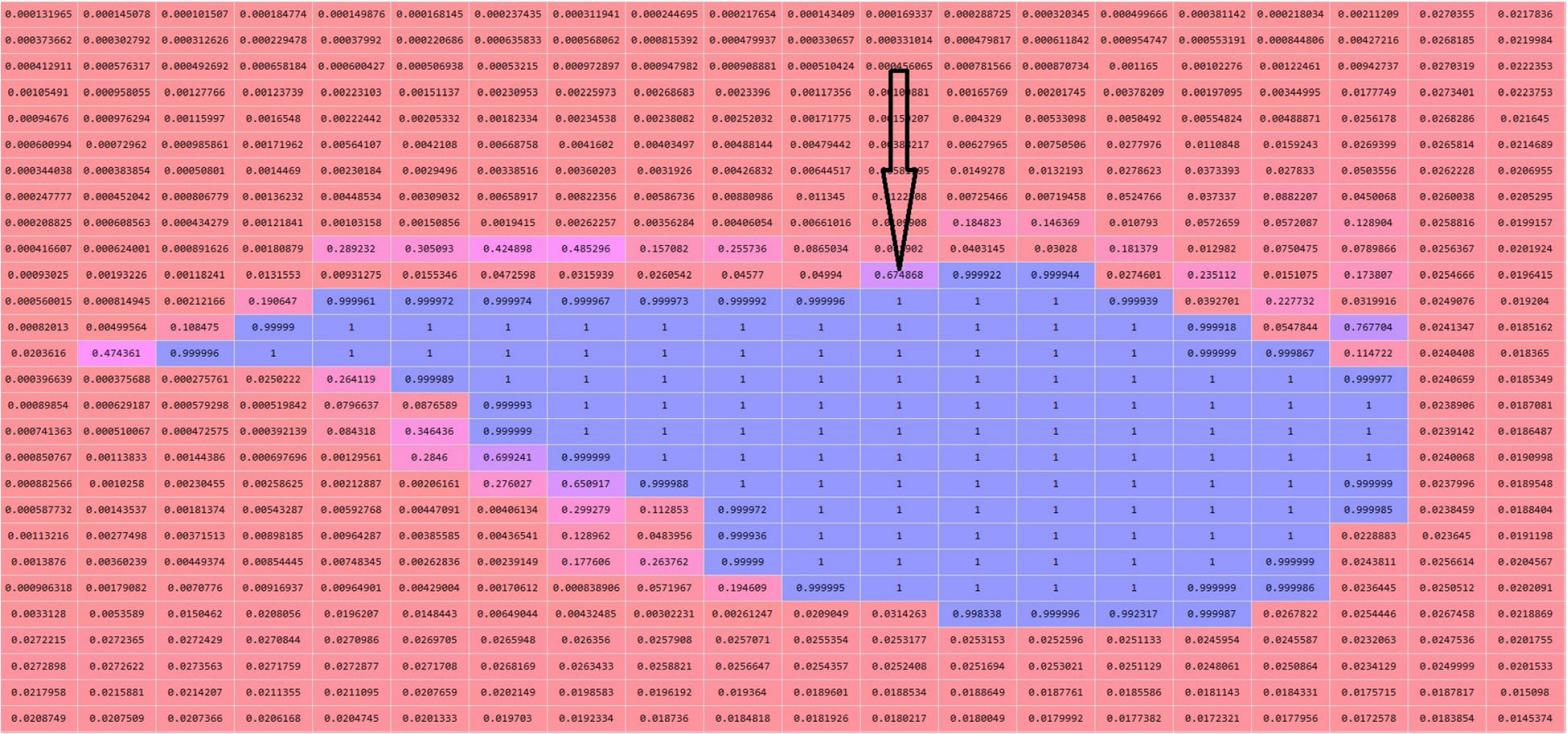
Graph of the prediction confidence matrix, where the arrow points to the predicted value of a single voxel point

Figure 3 presents the 3D segmentation prediction by the MV-SIR model; it shows the lung nodule cubes of the test set, the patches prepared point by point, and the recorded position of each voxel. The model predicts whether a patch is within a lung nodule, and the predicted value of each voxel is rearranged according to the position. Subsequently, the threshold image is binarized to obtain a mask of the segmented image; this mask is overlaid onto the original image to complete the pulmonary nodule and the 3D segmentation of the image. In this way, our MV-SIR model can obtain VH and SH of medical image features; their shallow, middle, and deep layer information; and information of different views for comprehensive judgment. Thus, effective improvement of image recognition and segmentation and enhancement of 3D segmentation accuracy can be realized.

### Evaluation

In order to evaluate the proposed model, the results predicted by the model are compared with the ground truth in terms of the metrics, the Dice coefficient, average surface distance (ASD), and Hausdorff distance (HSD). In addition, we measure the sensitivity (SEN) and positive predictive value (PPV) to determine the ability of the model to segment the ROI in the segmentation experiment. These metrics are calculated by the following formulas:

5 $$ \boldsymbol{DICE}=\frac{\mathbf{2}\ast \left({\boldsymbol{V}}_{\boldsymbol{seg}}\boldsymbol{and}\ {\boldsymbol{V}}_{\boldsymbol{gt}}\right)}{\left({\boldsymbol{V}}_{\boldsymbol{seg}}+{\boldsymbol{V}}_{\boldsymbol{gt}}\right)}, $$

6 $$ \boldsymbol{PPV}=\frac{\boldsymbol{V}\left(\boldsymbol{Gt}\cap \boldsymbol{Seg}\right)}{\boldsymbol{V}\left(\boldsymbol{Seg}\right)}, $$

7 $$ \boldsymbol{SEN}=\frac{\boldsymbol{V}\left(\boldsymbol{Gt}\cap \boldsymbol{Seg}\right)}{\boldsymbol{V}\left(\boldsymbol{Gt}\right)}, $$

8 $$ \boldsymbol{HSD}=\boldsymbol{\max}\left\{{\boldsymbol{\sup}}_{\boldsymbol{x}\boldsymbol{\epsilon } \boldsymbol{X}}{\boldsymbol{\operatorname{inf}}}_{\boldsymbol{y}\boldsymbol{\epsilon } \boldsymbol{Y}}\ \boldsymbol{d}\left(\boldsymbol{x},\boldsymbol{y}\right),{\boldsymbol{\sup}}_{\boldsymbol{y}\upepsilon \boldsymbol{Y}}{\boldsymbol{\operatorname{inf}}}_{\boldsymbol{x}\boldsymbol{\epsilon } \boldsymbol{X}}\ \boldsymbol{d}\left(\boldsymbol{x},\boldsymbol{y}\right)\right\}, $$

9 $$ \boldsymbol{ASD}=\frac{\mathbf{1}}{\mathbf{2}}\left({\boldsymbol{mean}}_{\boldsymbol{i}\boldsymbol{\epsilon } \boldsymbol{Gt}}{\boldsymbol{\min}}_{\boldsymbol{j}\boldsymbol{\epsilon } \boldsymbol{seg}}\ \boldsymbol{d}\left(\boldsymbol{x},\boldsymbol{y}\right)+{\boldsymbol{mean}}_{\boldsymbol{i}\boldsymbol{\epsilon } \boldsymbol{seg}}{\boldsymbol{\min}}_{\boldsymbol{j}\boldsymbol{\epsilon } \boldsymbol{Gt}}\boldsymbol{d}\left(\boldsymbol{x},\boldsymbol{y}\right)\right), $$

Here, ***V***_***gt***_ is the calibration ground truth, ***V***_***seg***_ is the model segmentation result, and ***x*** and ***y*** are the coordinates of the midpoint of the image, ***sup***_***xϵX***_***inf***_***yϵY***_**is** the shortest distance from a point in a point set to another point set, ***mean***_***iϵGt***_***min***_***jϵseg***_ is average of the closest distance between two points.

The software used for the implementation of the model is the Keras-gpu 2.2.4 platform developed by Google, and the hardware is Dell Workstation running on Windows®10, executed on Inter(R)Xeon(R) Gold 6130 CPU @2.10 GHZ (16 cores), with a 256 GB RAM, and a NVIDIA Quadro P5000 GPU.

## Result

### Learning curve

After 100 epochs of training, the MV-SIR model completely converged. The accuracy of the training set (ACC) and that of the verification set (Val_ACC) reached 99.10 and 98.91%, respectively. The loss of the training set (loss) and the verification set (Val_loss) decreased to 0.0321, and 0.0318, respectively.

**Fig. 4:**
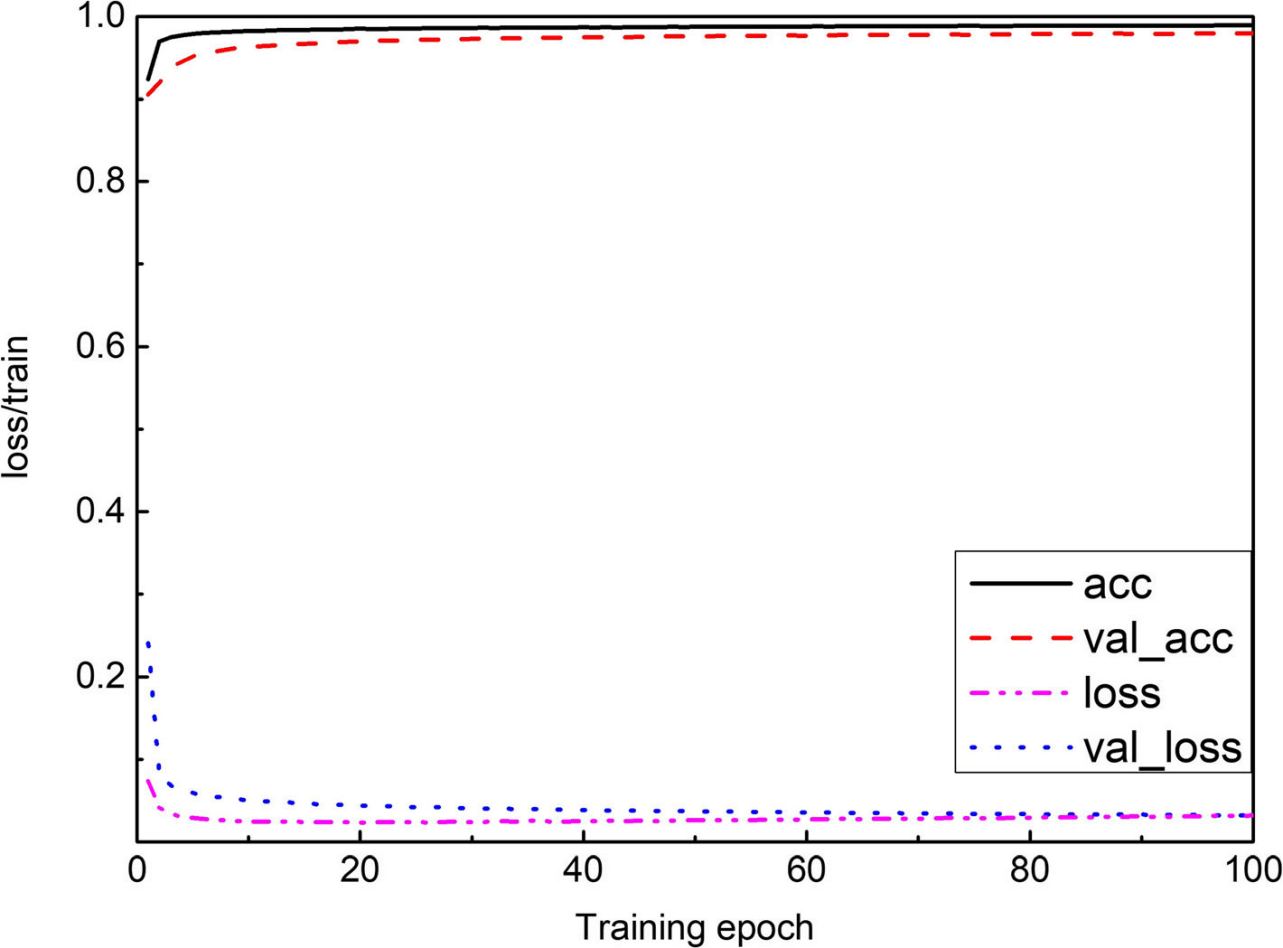
Learning curve of the MV-SIR model

Figure 4 indicates that the ACC values of the training set and the verification set increase rapidly 30 epochs after the training starts, then this increase rate reduces; finally, after 100 epochs, ACC remains constant. The same trend is observed for the loss values as well. These observations indicate that our MV-SIR model fully converges after 100 epochs, and a high ACC is achieved. The MV-SIR model generally takes only 2 h to complete the training process, its best performing training steps require only 100 epochs, and the prediction process is completed within 5 min; thus, the segmentation efficiency of the model is improved.

### Comparison of model structures

We analyzed the effect of different model structures on the 3D segmentation performance. For this analysis, we designed three model structures: the traditional multi-view input CNN (MV-CNN) model, the multi-view input residual block CNN (MV-I-CNN) model, and our MV-SIR model. The results indicate that with the improvement of the model structure, the 3D segmentation performance improves.

**Fig. 5:**
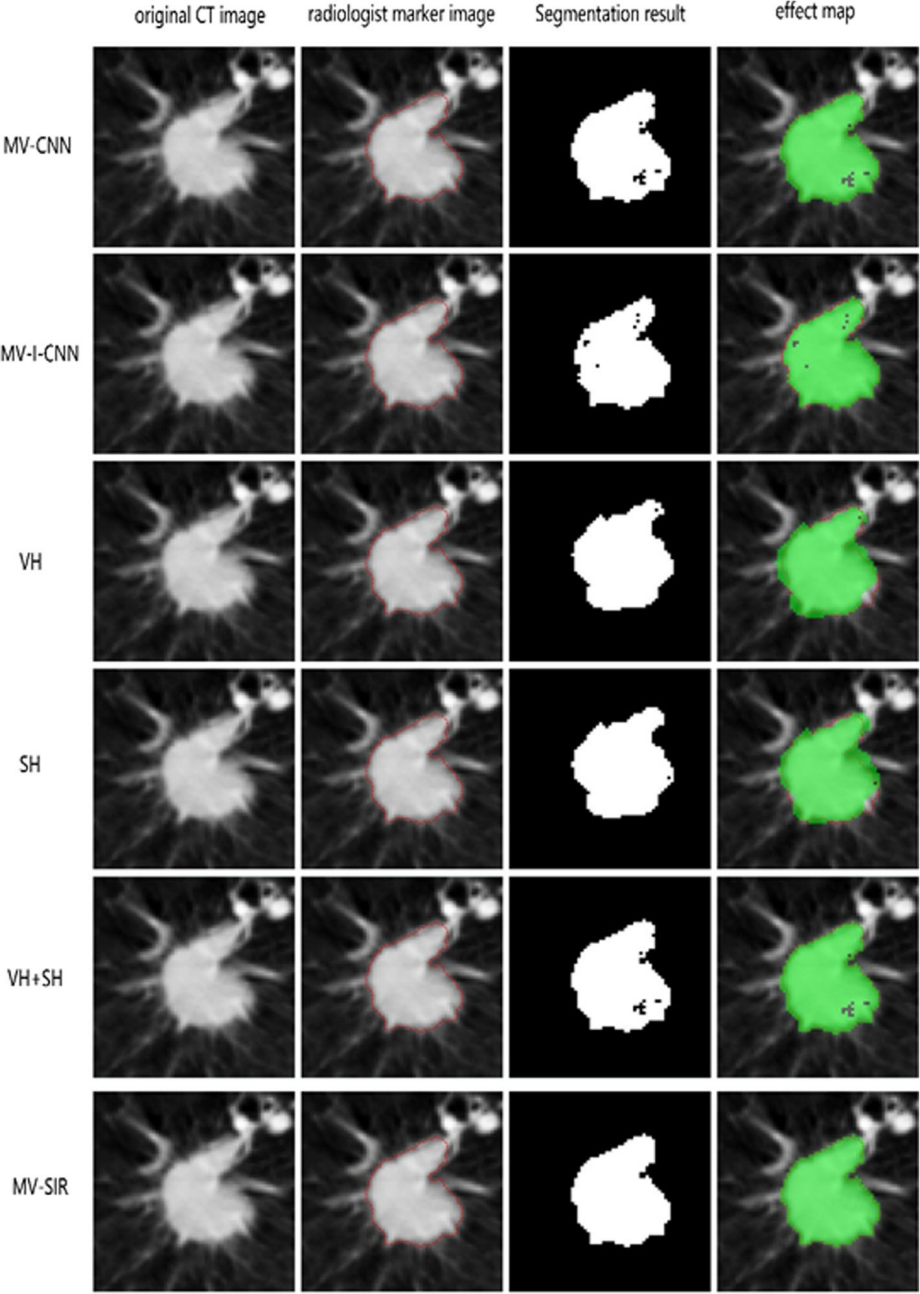
Comparison of model structures and different inputs. Columns from left to right are the original CT image, radiologist marker image, MV-SIR result, and 2D segmentation effect map. The top to bottom rows sequentially present the results of the MV-CNN, MV-I-CNN, the MV-SIR with VH input, the MV-SIR with SH input, the MV-SIR with combined VH and SH input, and secondary input MV-SIR. The 2D segmentation map can intuitively show that our model has achieved the best segmentation effect

Figure 5 presents the segmentation effect maps of the 2D slices obtained from the 3D segmentation results from the different models. Note that in the segmented lung nodule image predicted by the MV-CNN model, the internal pulmonary nodules are incomplete and the external image exceeds the lung nodule boundary, indicating the low accuracy of the model prediction and the presence of high false negatives and positives. The MV-I-CNN model performs better, but there are still a certain number of false positives. By contrast, our model achieves a satisfactory 3D segmentation effect. Table 1 presents the comparison of the 3D segmentation performances of our model and other models in terms of the metrics Dice, ASD, HSD, PPV, ACC, and SEN. The values indicate good performance of the MV-SIR model in terms of Dice, SEN compared to the other two models. In particular, the Dice value is 0.926, nearing the current high level in the 3D medical image segmentation industry and consistent with the result of 3D U-net medical image segmentation [28]; other parameters have a similar trend. In summary, we can conclude that the secondary input original image and residual block positively contribute to the improvement of model segmentation performance.

**Table 1:** Comparison of the 3D segmentation performances of different model structures

Models	ASD	HSD	Dice	PPV	SEN
MV-CNN	0.20 ± 0.030	2.78 ± 0.52	0.86 ± 0.18	0.939 ± 0.026	0.921 ± 0.095
MV-I-CNN	0.18 ± 0.07	4.80 ± 1.61	0.87 ± 0.03	0.917 ± 0.005	0.9620 ± 0.001
**MV-SIR**	**0.072 ± 0.033**	**1.293 ± 0.533**	**0.926 ± 0.035**	**0.936 ± 0.022**	**0.981 ± 0.113**

### Comparison of different inputs

Figure 5 also presents the comparison of the 3D segmentation performances of the MV-SIR model with different inputs, that is, VH features alone, SH features alone, VH and SH combined, and VH and SH combined along with the secondary input. It is noted that VH and SH together as input effectively improve the performance of medical image segmentation. Nevertheless, our secondary input model performs the best, indicating that the secondary input can significantly improve the 3D segmentation effect of the model.

In general, when the VH or SH features alone are input in the MV-SIR model, the image segmentation effect map in the 2D slice from the 3D segmentation result is incomplete, and the non-pulmonary nodules are identified as lung nodules. However, with VH and SH together as the input to the MV-SIR model, the apparent segmentation effect is considerably improved, with decreased false negatives and false positives of the predicted results. Moreover, the segmentation performance is the best in case of VH and SH together as the input along with the secondary input to the MV-SIR model.

Table 2 indicates that using VH or SH features alone as the MV-SIR model input results in the main disadvantage that more false positives appear in the prediction results. From Table 2, we can draw the following conclusions in terms of the Dice, ASD, HSD, PPV, and SEN indicators: the MV-SIR model performs the best in 3D segmentation, and the results of comparing different inputs prove that the secondary input can improve the accuracy of the model 3D segmentation.

**Table 2:** Comparison of the 3D segmentation performances of the model with different inputs

Models	ASD	HSD	Dice	PPV	SEN
VH	0.624 ± 0.242	7.79 ± 3.23	0.713 ± 0.196	0.914 ± 0.036	0.807 ± 0.106
SH	0.652 ± 0.043	4.632 ± 0.899	0.837 ± 0.034	0.953 ± 0.097	0.965 ± 0.028
VH + SH	0.422 ± 0.104	5.086 ± 1.968	0.889 ± 0.049	0.955 ± 0.005	0.972 ± 0.005
**MV-SIR**	**0.072 ± 0.033**	**1.293 ± 0.533**	**0.926 ± 0.035**	**0.936 ± 0.022**	**0.981 ± 0.113**

### Receiver operating characteristic curve (ROC) and model performance

To further confirm the effectiveness of our MV-SIR model in improving the 3D segmentation performance, we draw ROC curves for models with different inputs and different structures. In Section 3.4, we mentioned that in the process of model prediction and 3D reconstruction of segmentation, we need to choose an optimal threshold for binarization of reconstructed images. The ROC curve is a powerful tool to study the generalization performance of deep learning from the perspective of threshold selection. The value of the point closest to the upper left corner is the optimal threshold. The ROC curves for each model are plotted to the same coordinates to visually identify the pros and cons of the model. The model represented by the ROC curve near the upper left corner has the highest accuracy.

**Fig. 6:**
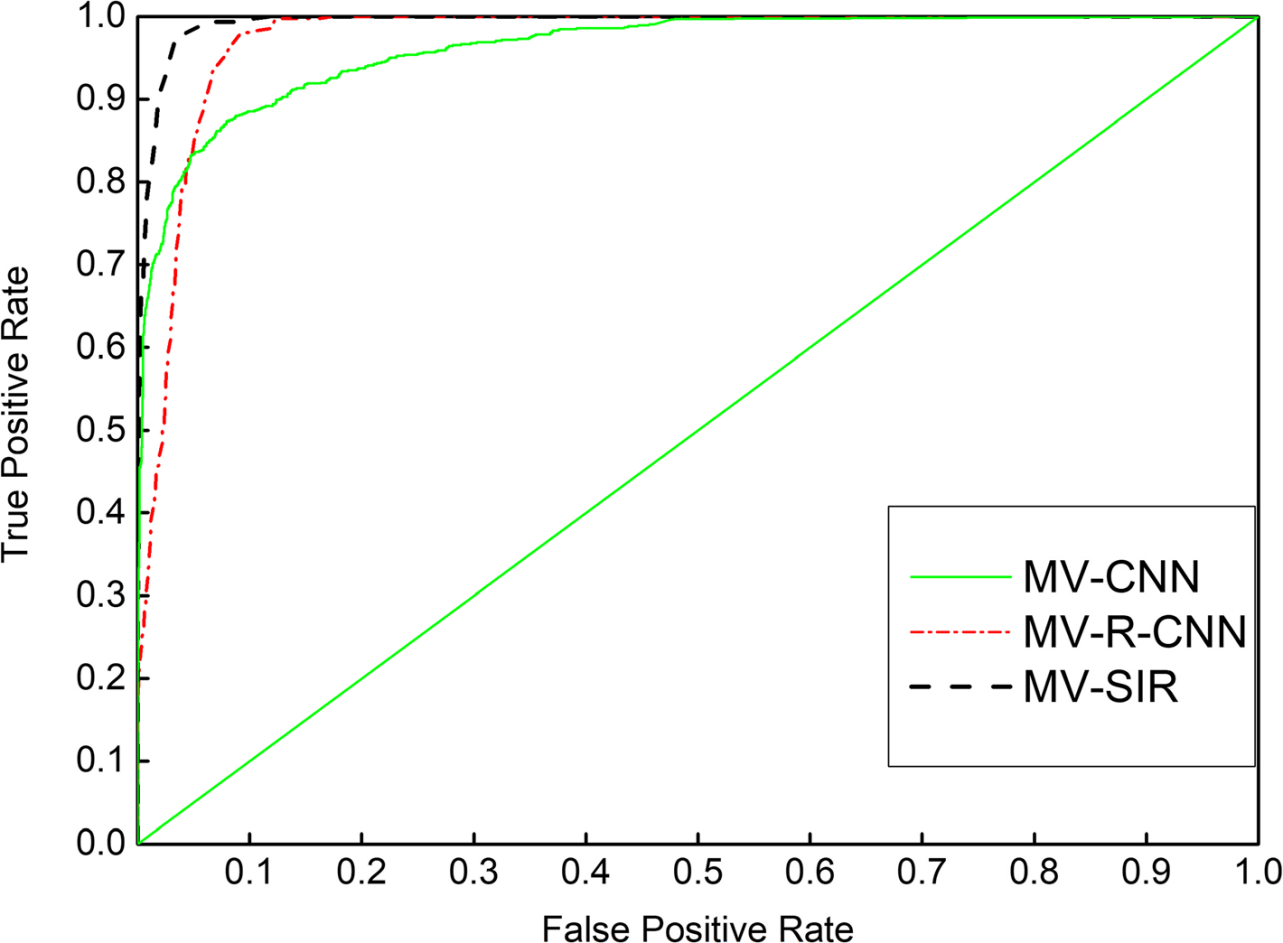
ROC curves of MV-CNN, MV-I-CNN, and MV-SIR

**Fig. 7:**
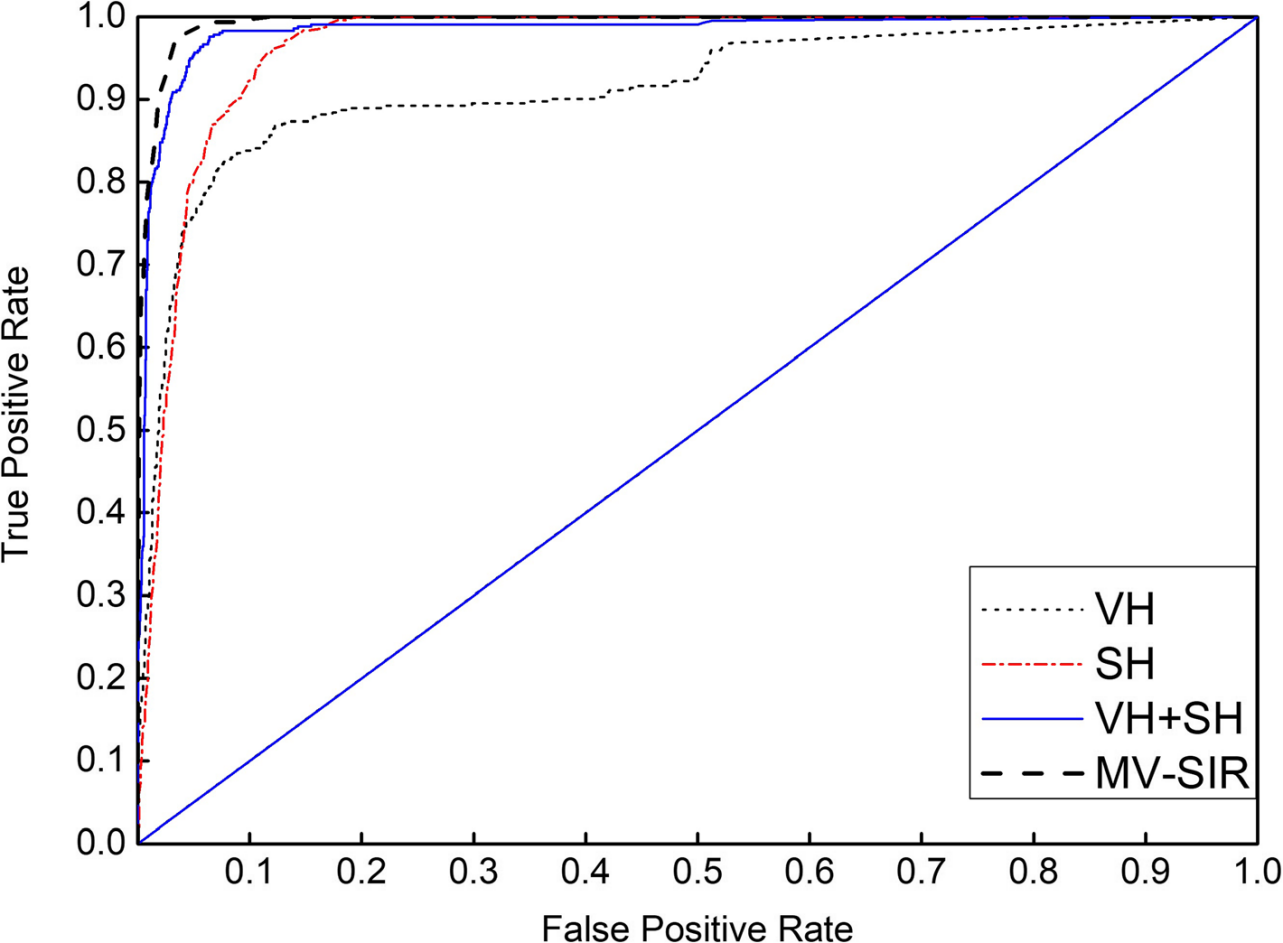
ROC curves of MV-SIR with different inputs, namely, VH, SH, VH and SH together, and the secondary input MV-SIR

Figures 6 and 7 present seven ROC curves drawn in two graphs, where the positive error ratio is the abscissa and the correct discipline is the ordinate. The figure indicates that the MV-SIR model performs better in 3D image segmentation than MV-CNN and MV-I-CNN. The optimal threshold of the MV-SIR model is less than those of the other two models. The result shows that the prediction results obtained by the MV-SIR model are relatively high. Figure 6 confirms the same conclusion that the MV-SIR model performs the best in medical image segmentation when considering the four different input models. In addition, the confidence of the prediction results obtained by the model is the highest.

Table 3 presents the results of the comparison of our model with other models. Our model achieves better performance in terms of Dice, SEN, PPV, HSD, and ASD. The Dice value of our model is comparable to that of the classic 3D U-net model, while other parameter values somewhat exceed the values of the U-net model.

**Table 3:** Comparison of the 3D segmentation performances of our model and other models

Models	ASD	HSD	Dice	PPV	SEN
Shahzad R [33]	1.553 ± 0.376	9.408 ± 3.059	0.885 ± 0.028	0.907 ± 0.052	0.867 ± 0.046
Tziritas G [34]	2.157 ± 0.503	19.723 ± 4.078	0.867 ± 0.047	0.861 ± 0.062	0.889 ± 0.108
**U-net** [28]	0.940 ± 0.193	8.628 ± 3.390	0.926 ± 0.016	0.940 ± 0.028	0.916 ± 0.048
Roth HR [17]	0.240 ± 0.330	15.086 ± 1.968	0.776 ± 0.157	0.775 ± 0.158	0.837 ± 0.207
Zeng G [35]	1.050 ± 0.360	11.730 ± 6.620	0.905 ± 0.028	0.912 ± 0.168	0.897 ± 0.106
**MV-SIR**	**0.072 ± 0.033**	**1.293 ± 0.533**	**0.926 ± 0.035**	**0.936 ± 0.022**	**0.981 ± 0.113**

**Fig. 8:**
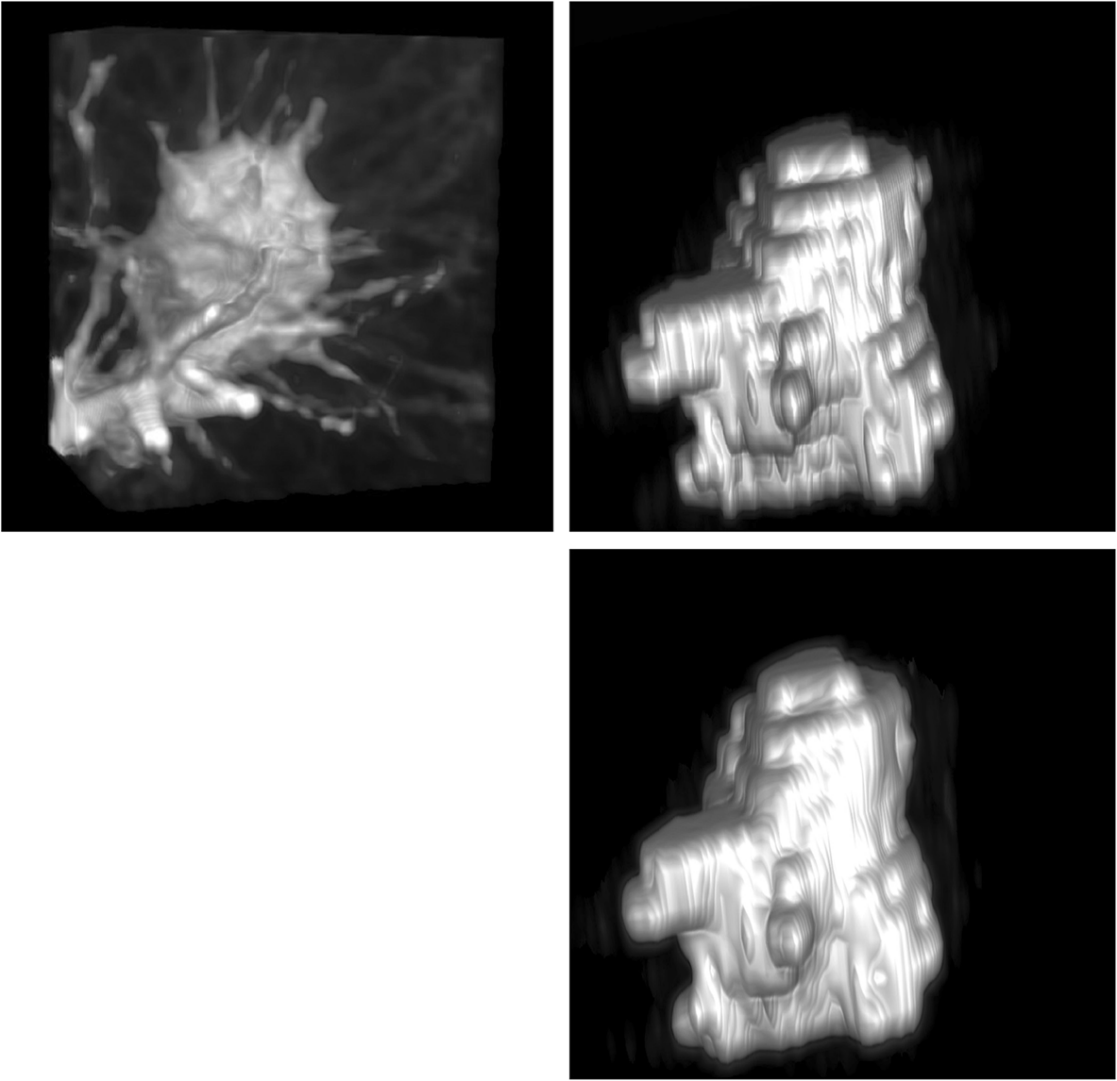
MV-SIR model 3D segmentation result. Top left: the lung nodule original image; top right: ground truth (GT) map of expert calibration; and bottom: the MV-SIR prediction map

Figure 8 presents the results of 3D reconstruction of the original CT image, the GT map of the expert calibration, and the prediction map of our model. The 3D segmentation predicted by our model is very close to the GT map of the expert calibration, which intuitively implies that our model has achieved superior results in 3D segmentation of pulmonary nodules.

We use the QIN LUNG CT public data set to test our model. The computed tomography (CT) image data of this data set comes from patients diagnosed with non-small cell lung cancer (NSCLC). We are very pleased to see that our model has an average DICE of 0.920 ± 0.027 in 47 cases on this dataset. It shows that our model has good segmentation results for different data sets in the segmentation of lung nodules.

## Discussion

3D medical image segmentation has always been a challenging task. Our goal is to improve the accuracy and confidence of 3D medical image segmentation to assist physicians in clinical diagnosis and treatment. In this study, we proposed the MV-SIR model to improve the performance of medical image 3D segmentation.

By presenting the MV-SIR with color scale patches extracted around a particular pixel, the CNN can simply be used to classify each pixel in the image [4]. We extracted the characteristic patches from three perspectives, namely, axial, coronal, and sagittal views, in the lung nodule cube. Multi-view patches help improve image quality and anatomy and extend the field of view [36].

For each patch, we further extracted VH and SH features. As shown in Fig. 1 VH can predominantly learn grayscale value information, whereas SH can predominantly learn boundary information when they are used separately as the input to the model. By combining them as the input to the model, the model learns greater patch information and thus gains more sense of vision. To validate this concept, we compared the performance of the MV-SIR model under different inputs, namely VH, SH, and VH and SH together. We found that VH and SH together as the input to the model yields greatly improved Dice, HSD, SEN, and other parameter values. In addition, the ROC curve clearly indicates the superior 3D segmentation result obtained by the model with the combined VH and SH input.

We believe that multi-view and VH and SH feature maps together as the input yield improved 3D segmentation performance of the model mainly because the model can extract more information of the image, fields of view, and boundaries as well as pixel values, producing excellent mutual effect. This conclusion is consistent with the previous studies [37–39].

The difference in the layers of the network implies that different features of different levels can be extracted [40, 41]. The more layers of the network, the deeper feature information extracted from the image. In the proposed model, we include a residual block to the traditional CNN; we skip the three-layer convolution by connecting the input information of P2 and calculate the output of the residual block ***F(x) + x***. This process results in the two matrices being added, with the dimensions of the matrix unchanged. This implies that we add the feature information of the first two layers directly to the subsequent output, and the value of the matrix changes. Characteristic information of different levels can be extracted to a certain extent. We add another residual block to the network and input the image the second time, but in another path. Then, after only one convolution and pooling, the obtained feature matrix is ​​spliced ​​to the fast residual output. Note that we use a matrix splicing function, where the values ​​in the matrix remain unchanged, while the size of the matrix becomes twice as large.

Different characteristics of different network layers can be obtained at the final fully connected layer. The shallow information and the deep information are used together as the basis for judgment in our 3D image segmentation, thus improving the performance of 3D segmentation by our model.

Further, we designed three network structure models: MV-CNN, MV-I-CNN, and MV-SIR. The results of segmentation indicators such as Dice, HSD, SEN, and segmentation rendering all indicate that the segmentation effect of the proposed model is superior, in turn, confirming the validity of our concepts based on which the model has been designed. Moreover, the ROC curves obtained from our model and the previous models as well as our model with different inputs confirm that the MV-SIR model achieves superior performance in 3D medical image segmentation. In future work, we hope to design a network model with multiple iterations to further validate our concepts.

One challenge is how to apply our model to real-world CT images. we hope to expand the cube containing lung nodules to a whole 3D volume. In the training process, a larger amount of calculation is required to complete the automatic 3D segmentation of the whole 3D volume. Another feasible solution is that the doctor calibrates the position of the lung nodule cube to assist our model in 3D segmentation.

## Conclusion

In this study, we provide a well-structured deep learning model MV-SIR for 3D segmentation of pulmonary nodules. Our model consists of six SIR submodels, each of which adds two fast residual blocks and one secondary input module to the traditional CNN. From the LIDC-IDRI dataset, 19 million patches were extracted from 600 lung nodules used for model training and 274 lung nodules used for model testing. The test results indicate that the MV-SIR model achieved excellent performance in 3D pulmonary nodule segmentation, with a Dice of 0.926 and an ASD of 0.072. In future work, we plan to include more repeated inputs in the model, and test the segmentation performance of the MV-SIR model on different datasets.

## Data Availability

The dataset(s) supporting the conclusions of this article is (are) available in the public Research Data Deposit platform (https://www.cancerimagingarchive.net/).

## Abbreviations

CNNs: Convolutional neural networks
2D: Two-dimensional
3D: Three-dimensional
MV-SIR: Multi-view secondary input residual
LIDC-IDRI: Lung image database consortium and image database resource initiative
CT: Computed tomography
MRI: Magnetic resonance imaging
VH: Voxel heterogeneity
SH: Shape heterogeneity
SIR: Secondary input residual
FNN: Fuzzy neural network
ROI: Region-of-interest
ASD: Average surface distance (ASD)
HSD: Hausdorff distance
SEN: Sensitivity
PPV: positive predictive value
ACC: Accuracy of the training set
Val_ACC: Accuracy of the verification set
loss: The loss of the training set
Val_loss: The loss of the verification set
MV-CNN: The traditional multi-view input CNN
MV-I-CNN: The multi-view input residual block CNN
ROC: Receiver operating characteristic curve
GT: Ground truth
